# A large single-center prospective study to investigate the factors influencing the choice of breast-conserving surgery versus mastectomy in Chinese women with early breast cancer

**DOI:** 10.1186/s12957-023-02924-y

**Published:** 2023-02-11

**Authors:** Zhensheng Li, Yunjiang Liu, Jing Zhang, Yue Li, Kaiye Du, Shuo Zhang, Huina Han, Jun Zhang

**Affiliations:** 1 Department of Radiation Oncology, the Fourth Hospital of Hebei Medical University, Shijiazhuang, 050035 China; 2 Department of Breast Surgery, the Fourth Hospital of Hebei Medical University, Shijiazhuang, 050035 China

**Keywords:** Early breast cancer, Breast-conserving surgery, Mastectomy, Decision-making, Chinese

## Abstract

**Background:**

Compared to mastectomy, breast-conserving surgery (BCS) provides the same survival rate and a higher quality of life for patients with early breast cancer (EBC). However, Chinese women with EBC are known to have a low BCS rate. A large prospective cohort study was conducted to investigate the factors influencing the choice of BCS in this population.

**Methods:**

In 2017, all women with unilateral EBC and eligible for BCS at our institution were enrolled. Before surgery, the patient’s trust in the surgeon and her perceived strength of the surgeon’s recommendation of BCS were measured through an in-person interview and validated ad hoc questionnaire. Multivariate logistic regressions on BCS procedure *vs.* mastectomy were used to estimate the odds ratio (OR).

**Results:**

One thousand one hundred thirty-six patients enrolled at analysis had an average age of 51.8 and tumor size of 2.4 cm. 19.9% of patients had BCS. The “*strong*” level of trust in the surgeon was significantly associated with BCS with an OR of 2.944 (*p*<0.001) when compared to the “*average or under*” trust. The “*strong*” and “*moderate*” strengths in surgeon recommendation for BCS were also found to be significantly associated with the BCS procedure with ORs of 12.376 (*p* <0.001) and 1.757 (*p* =0.040), respectively, compared to the “*neutral or dissuaded*” strength.

**Conclusions:**

Stronger trust in surgeons and BCS recommendation by surgeons are associated with a higher rate of BCS in Chinese women with EBC. Interventional trials are needed to confirm this finding.

## Introduction

It is well-established that breast-conserving surgery (BCS) followed by radiotherapy (RT) provides the same survival rate and higher quality of life (QoL) compared to a mastectomy for patients with early breast cancer (EBC) [1–4]. While the rate of BCS in the US has consistently been 60–70% for many years, the rate of BCS in mainland China is substantially lower at 10–20% [4, 5]. Even outside of China, Chinese patients in Hong Kong, Southeast Asia, and the USA have lower rates of BCS compared to patients of other races [5–7]. Many interrelated factors such as age, education level, disease stage, RT cost after BCS, cultural beliefs, and surgeon-patient relationship have been identified to influence the BCS choice [8–13]. By promoting BCS among Chinese women with EBC, their QoL would be improved. As a result, greater efforts should be made to identify the possible race-related factors, especially the actionable ones that influence the BCS choice [14–16]. In 2016, we initiated a prospective cohort study to investigate the low BCS phenomenon at a large cancer center in mainland China. In this study, we hypothesized that actionable factors like the amount of trust in the surgeon and the surgeon’s recommendation greatly influence Chinese women with newly diagnosed EBC to prefer BCS over mastectomy.

## Materials and methods

### Study design

All women with newly diagnosed EBC at our institution and who were eligible for BCS in 2017 were prospectively enrolled. Inclusion criteria included (1) a pathological diagnosis age of 18–75 years old, (2) unilateral breast cancer, (3) a tumor size in a conservable breast with an acceptable cosmetic outcome, and (4) basic communication capability in Chinese during the interview. The third criterion was decided by two private investigators who considered the percentage of the tumor size in the breast volume that was removed, along with the tumor location. A physical examination, mammogram, ultrasound, and magnetic resonance imaging (MRI) of the breast were used to determine the BCS eligibility. Exclusion criteria included (1) inflammatory or T4 stage tumor, (2) more than one tumor in different quadrants, (3) diffuse cancerous microcalcifications, and (4) contraindications to postoperative RT such as pregnancy, history of chest RT, and/or collagen vascular diseases. All participating surgeons had at least 10 years of surgical experience in BCS, mastectomy, and other related procedures including oncoplastic breast surgery. Neoadjuvant chemotherapy (NAC), sentinel lymph node biopsy (SLNB), and axillary lymph node dissection (ALND) procedures were not listed as exclusion criteria. The patient’s residential area was classified as urban *vs*. rural based on geographic data. Our institution is located at the center of the urban region.

In the analysis, the surgery date was defined as the baseline. Having or not having the BCS was defined as the study endpoint. The patient’s choice of having BCS was not established as the analysis endpoint because the BCS eligibility could have suddenly been denied by a positive SLNB result on the date of surgery. The surgeon would know the patient’s choice of BCS *vs*. mastectomy at a private meeting after providing the BCS-related cosmetic and psychological gain, similar tumor control and survival, and additional cost of RT. Handwritten figures of breasts could be presented for specific explanation. To account for different levels of experience among the surgeons in the center, a BCS Composite Index (BCS-CI) score was assigned to each surgeon. The score was estimated from the number of BCS procedures performed by the surgeon in 2016 among those EBC patients who were eligible for BCS on the same criteria as this study. The BCS-CI score presumably served to historically summarize the surgeon’s general attitude, experience, and use of BCS for his/her patients. BCS-CI scores were divided into three levels: low, medium, or high. Each surgeon was given one of those three score levels during analysis.

The study was approved by the Medical Research Ethics Committee of the Fourth Hospital of Hebei Medical University in 2016 (record #: 2016-026). A signed informed consent from all study participants was obtained before any study procedure started. The study was registered as the “ChiCTR-RRC-17011662” in the Chinese Clinical Trial Registry (http://www.chictr.org.cn).

### Questionnaires and Interview

Each patient had one in-person interview with a well-trained researcher one day before her scheduled surgery date. While no clinical staff members were allowed to be on site, one patient caretaker could be present for dialect translations or explanations when necessary. After the patient signed the study’s declaration statement on anonymity and confidentiality, she was instructed to fill out the ad hoc questionnaire designed for the study. Prior to the study initiation, a pilot study was conducted to validate the questionnaire with satisfactory performance on the feasibility and validity of its content and construct in a small population of patients (*n* =22). Questions in the questionnaire are translated from Chinese to English and are shown below.

**Table:** 

Question number	Question
1	What is the highest level of education the patient achieved?
2	What is the level of trust the patient has in her surgeon?
3	Has the patient heard about the BCS procedure before?
4	Did the surgeon recommend the BCS procedure?
5	If yes to Question #4, how strong did the patient perceive her surgeon’s recommendation to be?
6	How does the additional cost of 25,000 RMB for the required RT after BCS influence the patient’s choice of surgery?

More specifically, the choice of questions was for the level of trust — (a) *extremely strong*, (b) *strong*, (c) *average*, and (d) *mild or lower*; for the strength of BCS recommendation — (a) *very strong*, (b) *strong*, (c) *moderate*, and (d) *no or dissuaded*; and for the BCS influence by RT cost — (a) *yes and very much*, (b) *yes and somewhat*, and (c) *not at all.* No independent assessor was used in the interview. The consistency of recommendation strengths assessed by the patient and surgeon could not be investigated due to the lack of a surgeon note that could be evaluated. One-third of the charts did not have this information.

### Statistical analysis

Descriptive statistics were presented with a mean or percentage, median, and standard deviation (SD). Comparisons were conducted with ANOVA or Chi-squared tests. Logistic linear regression models were used to estimate the odds ratio (OR) and 95% confidence interval (CI) with a *p* value. The final multivariate model and its covariates were determined after the full examination of univariate analysis results, literature review, and a stepwise model building process. A two-sided *p*<0.05 was considered statistically significant. All statistical analyses were performed with SAS 9.4.

## Results

### Patient characteristics

A total of 1191 women were enrolled in the study. 17 (1.4%) patients refused the scheduled interview. Of the remaining 1174 women, 38 (2.7%) patients had voluntarily switched from BCS to mastectomy after finding riskier pathological results from SLNB or other new reports. In the end, 1136 women were analyzed. They had an average age of 51.8 ± 10.3 (25–75) years and an average tumor size of 2.4 ± 0.9 (0.3–6.0) cm. 64.7% (735/1136) patients reported the BCS being mentioned or introduced from the surgeon. Overall, 19.9% of patients had BCS (Table 1).

**Table 1:** Patient and tumor characteristics of the study population

Variable		Surgery choice			
		Mastectomy	BCS	*p*^a^	All
Patients		910 (*80.1*)	226 (*19.9*)		1136 (100%)
Residence	Urban	306 (*69.7*)	133 (*30.3*)	*<0.001*	439 (*38.6*)
	Rural	604 (*86.7*)	93 (*13.3*)		697 (*61.4*)
Age (years)	≤40	108 (*70.1*)	46 (*29.9*)	*<0.001*	154 (*13.6*)
	41-50	282 (*74.1*)	99 (*26.0*)		381 (*33.5*)
	51-60	290 (*84.3*)	54 (*15.7*)		344 (*30.3*)
	≥61	230 (*89.5*)	27 (*10.5*)		257 (*22.6*)
Surgeon BCS-CI*	Low	521 (*87.6*)	74 (*12.4*)	*<0.001*	595 (*52.4*)
	Medium	319 (*76.9*)	96 (*23.1*)		415 (*36.5*)
	High	70 (*55.6*)	56 (*44.4*)		126 (*11.1*)
Tumor laterality	Right	457 (*78.3*)	127 (*21.7*)	*0.108*	584 (*51.4*)
	Left	453 (*82.1*)	99 (*17.9*)		552 (*48.6*)
Tumor size (cm)	<2.0	144 (*67.9*)	68 (*32.1*)	*<0.001*	212 (*18.7*)
	2.0–2.9	337 (*78.7*)	91 (*21.3*)		428 (*37.7*)
	≥3.0	367 (*89.5*)	43 (*10.5*)		410 (*36.1*)
	No record	62 (*72.1*)	24 (*27.9*)		86 (*7.6*)
Pathology	DCIS	61 (*70.9*)	25 (*29.1*)	*0.027*	86 (*7.6*)
	Invasive	849 (*80.9*)	201 (*19.1*)		1050 (*92.4*)
NAC	Not	884 (*80.5*)	214 (*19.5*)	*0.067*	1098 (*96.7*)
	Yes	26 (*68.4*)	12 (*31.6*)		38 (*3.3*)
ALND LN status	Not done	350 (*66.7*)	175 (*33.7*)	*<0.001*	525 (*46.2*)
	Positive	245 (*92.1*)	21 ( *7.9*)		266 (*23.4*)
	Negative	315 (*91.3*)	30 ( *8.7*)		345 (*30.4*)
SLNB LN status	Not done	400 (*93.5*)	28 ( *6.5*)	*<0.001*	428 (*37.7*)
	Positive	75 (*69.4*)	33 (*30.6*)		108 (*9.5*)
	Negative	435 (*72.5*)	165 (*27.5*)		600 (*52.8*)
Education	Middle and under	639 (*87.9*)	88 (*12.1*)	*<0.001*	727 (*64.0*)
	High school	154 (*69.7*)	67 (*30.3*)		221 (*19.5*)
	College and above	117 (*62.2*)	71 (*37.8*)		188 (*16.5*)
Trust on surgeon	Average and under	638 (*88.9*)	80 (*11.1*)	*<0.001*	718 (*63.2*)
	Strong	272 (*65.1*)	146 (*34.9*)		418 (*36.8*)
Patient heard BCS	No	459 (*85.3*)	79 (*14.7*)	*<0.001*	538 (*47.4*)
	Yes	451 (*75.4*)	147 (*24.6*)		598 (*52.6*)
Surgeon mentioned BCS	Not	375 (*93.5*)	26 (*6.5*)	*<0.001*	401 (*35.3*)
	Yes	535 (*72.8*)	200 (*27.2*)		735 (*64.7*)
Strength of BCS recommendation	Neutral/dissuaded	481 (*94.1*)	30 (*5.9*)	*<0.001*	511 (*45.0*)
	Moderate	282 (*88.7*)	36 (*11.3*)		318 (*28.0*)
	Strong	147 (*47.9*)	160 (*52.1*)	*<0.001*	307 (*27.0*)

*BCS* breast-conserving surgery, BCS-CI* BCS Composite Index, *DCIS* ductal carcinoma in situ, *NAC* neoadjuvant chemotherapy, *ALND* axillary lymph node dissection, *LN* lymph node, *SLNB* sentinel lymph node biopsy

*Defined as surgeon’s tertile level based on individual BCS rate performed in 2016

^a^*p* value from chi-squared test

Patients who had BCS were younger, more highly educated, living in urban regions, and had smaller tumor sizes (2.0 ± 0.8 cm) compared to patients who had a mastectomy. Not surprisingly, the BCS procedure was clinically and selectively related to the application of NAC, SLNB, and non-ALND under the institution’s guideline then. The BCS seemed significantly associated with the trust in the surgeon, BCS introduction from the surgeon, and BCS recommendation strength from the surgeon (all *p*<0.001). Multivariate logistic regression was required to evaluate the independence of these relationships.

### Logistic regression analysis

We excluded Question #6 from the analysis because of its low (19%) response rate. Table 2 shows the analysis results of the final model. The variable “*if surgeon introduced BCS*” was ultimately excluded in the final model because of its significant collinearity with the strength of BCS recommendation. The “*strong*” and “*moderate*” strengths of BCS recommendation were significantly associated with the BCS procedure with ORs of 12.376 (*p*<0.001) and 1.757 (*p*=0.040), respectively, compared to a “*neutral or dissuaded*” strength. Patients with tumor sizes <2.0 and 2.0–2.9 cm were also significantly associated with more likely having BCS with ORs of 3.792 (*p*<0.001) and 2.260 (*p*=0.001), compared to patients with tumor sizes ≥3.0 cm. A “*strong*” trust in the surgeon was significantly associated with the BCS procedure with an OR of 2.944 (*p*<0.001), compared to the “*average and lower*” trust level. Lastly, the “*high school*” and “*college and up*” education levels were closely twice as likely to be associated with the BCS procedure, compared to a “*middle and under*” education level.

**Table 2:** Univariate and multivariable logistic regression on the BCS procedure versus mastectomy

Variable			Univariate			Multivariate ^a^	
		OR	95%CI	*p*	OR	95%CI	*p*
Residence	Urban	2.823	(2.094–3.805)	*<0.001*	1.486	(0.982–2.247)	*0.061*
	Rural	1.000		*ref.*	1.000		*ref.*
Age (years)	≤40	3.628	(2.141–6.147)	*<0.001*	1.504	(0.769–2.941)	*0.233*
	41–50	2.990	(1.888–4.736)	*<0.001*	1.327	(0.752–2.339)	*0.329*
	51–60	1.586	(0.969–2.598)	*0.067*	0.858	(0.468–1.572)	*0.619*
	≥61	1.000		*ref.*	1.000		*ref.*
Tumor size (cm)	<2.0	4.030	(2.628–6.182)	*<0.001*	3.792	(2.247–6.399)	*<0.001*
	2.0–2.9	2.305	(1.558–3.410)	*<0.001*	2.260	(1.420–3.598)	*0.001*
	≥3.0	1.000		*ref.*	1.000		*ref.*
	No record	3.304	(1.873–5.826)	*<0.001*	1.948	(0.976–3.887)	*0.059*
Pathology	DCIS	1.731	(1.060–2.826)	*0.028*	1.388	(0.717–2.689)	*0.331*
	Invasive	1.000		*ref.*	1.000		*ref.*
Education	Middle and under	1.000		*ref.*	1.000		*ref.*
	High school	3.159	(2.197–4.543)	*<0.001*	1.875	(1.185–2.967)	*0.007*
	College and above	4.406	(3.045–6.377)	*<0.001*	1.776	(1.068–2.953)	*0.027*
Trust on surgeon	Average and under	1.000		*ref.*	1.000		*ref.*
	Strong	4.281	(3.148–5.821)	*<0.001*	2.944	(2.041–4.245)	*<0.001*
Strength of BCS recommendation	Neutral/dissuaded	1.000		*ref.*	1.000		*ref.*
	Moderate	2.047	(1.234–3.396)	*0.006*	1.757	(1.027–3.006)	*0.040*
	Strong	17.451	(11.335–26.867)	*<0.001*	12.376	(7.728–19.819)	*<0.001*

*BCS* Breast-conserving surgery, *OR* odds ratio, *CI* confidence interval, *ref.* reference

^a^The final multivariable model was established by using the purposeful stepwise strategy on all characteristics listed in Table 1

### Stratified logistic regression analysis

**Fig. 1:**
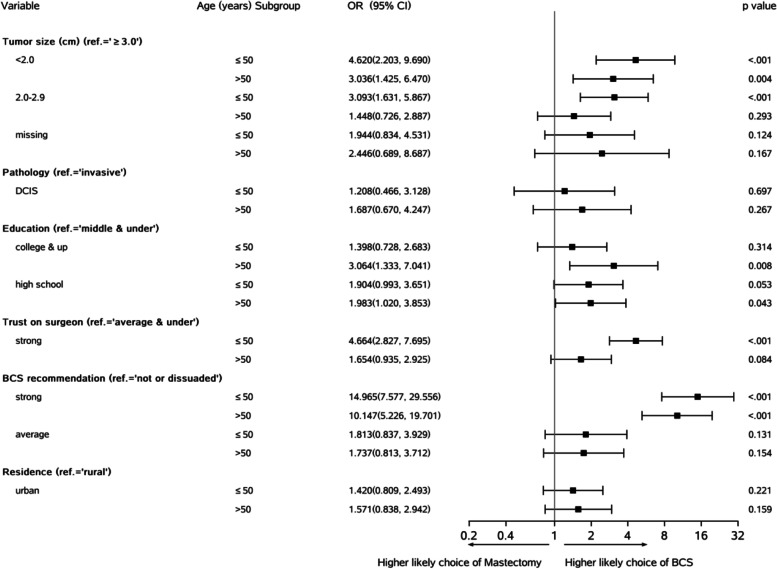
Results of multivariate logistic regression analyses stratified by age ≤50 (*N* =535) and age > 50 (*N* =601) subgroups. Covariates included all characteristics listed in Table 2 except the 4-level age group

Given the clinical importance and non-actionable feature, age (≤50 *vs*. >50 years) and resident status were considered as two stratified factors. Figure 1 shows that at age subgroups, the “*strong*” (*vs.* “*neutral or dissuaded*”) recommendation and tumor size <2.0 cm (*vs*. “*≥* 3cm”) were the most significant variables relating to BCS (OR =3.036–14.965, *p*<0.001). In younger patients (age ≤50 years), the moderate size tumor (“2.0–2.9” *vs*. “≥3” cm) was still associated with the BCS procedure (OR =3.093, *p*<0.001). This suggests that younger patients were more open to the BCS compared to older patients. Similarly, the “*strong*” trust on the surgeon (*vs*. “*average and under*”) in younger patients was more likely to be associated with the BCS procedure (OR =4.664, *p*<0.001) than it was in older patients (OR=1.654, *p*=0.084). There was a significant effect (OR =3.064, *p*=0.008) of “*college and higher*” (*vs*. “*middle and under*”) education in older patients on the choice for BCS. The “*strong*” (*vs*. “*neutral or dissuaded*”) strength of BCS recommendation remained high and significantly associated with the BCS procedure (OR=10.147–14.965, *p*<0.001) in both age subgroups. In both age subgroups, residence status (“*urban*” vs. “*rural*”) had no effect on the BCS procedure (*p*=0.159–1.221).

**Fig. 2:**
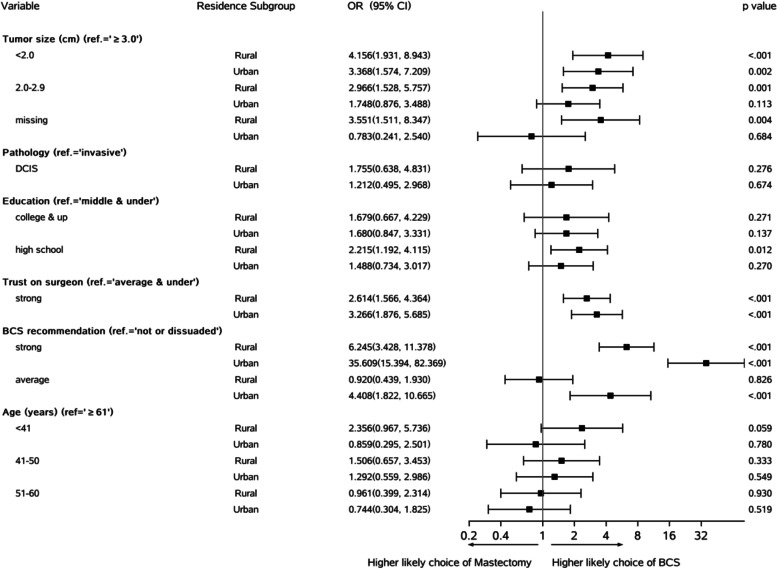
Results of multivariate logistic regression analyses stratified by “*rural*” (*N* =697) and “*urban*” (*N* =439) subgroups. Covariates included all characteristics listed in Table 2 except the residence status

Figure 2 shows that compared to “≥3 cm,” tumor size “2.0–2.9 cm” was significantly associated with the BCS procedure in the “*rural*” region (OR =2.966, *p*=0.001) but not in the “*urban*” region (OR =1.748, *p*=0.113). The smaller sample sizes of “urban” patients could explain the weaker or lack of relationship with the BCS procedure. In both residence subgroups, the “*strong*” trust (*vs*. “*average and under*”) on the surgeon was significantly associated with BCS (OR=2.614–3.266, *p*<0.001). Similarly, the “*strong*” recommendation (*vs.* “*neutral/dissuaded*”) was also significantly associated with BCS (OR =6.245–35.609, *p*<0.001) in both residence subgroups. However, the “*average*” recommendation (*vs*. “*neutral or dissuaded*”) seemed to be significantly associated with BCS only in the “urban” patients (OR =4.408, *p*<0.001).

## Discussion

The exact reasons why Chinese women with EBC are associated with a low rate of BCS worldwide remains largely unknown [5, 7]. While studies have demonstrated that unique ethnic or sociocultural features could play some roles, it is critical to identify those actionable factors in order to effectively promote BCS among Chinese patients [10]. Also, it would be better to have the quantitative profiles of those actionable factors [5]. In this prospective study, we found that smaller tumor size, especially one with a mean size of less than 2 cm, would significantly increase the BCS rate in Chinese women with EBC. We also found that the patient’s education level, rather than their age or residence, is independently correlated with the BCS choice. As a result, surgeons should particularly be careful and detailed in knowing these variable values when recommending BCS to a patient. Also, no matter what education level the patient has, the patient is more aware of and better understands the benefits and process of BCS. Lastly and most importantly, we found that the stronger “*trust on surgeon*” and “*BCS recommendation*” perceived by patients are very significantly associated with the BCS procedure, regardless of other traditional influencing factors. Overall, these findings would help optimize the efforts of surgeon teams to promote the BCS choice among Chinese women with EBC.

Many factors are involved when choosing to have BCS [6, 10]. It is challenging to quantitatively characterize the effects of patient beliefs or patient-surgeon dialog on the BCS choice. A previous study found the patients’ awareness of “*no difference in survival*” was recognized as the most influential factor [17]. The “*fear of cancer recurrence*” and “*removing the entire breast to gain peace of mind*” were found to be correlated with women choosing a mastectomy [16, 18]. Other factors like “*body image*,” “*physical appearance*,” being “*less disfiguring*,” the “*importance of breast to sexuality*,” and “*femininity*” were also found to be positively related to the BCS choice [10]. Meanwhile, categories like “*avoiding RT*,” “*living in remote areas*,” or “*employment related*” were found to be associated with the increased choice of mastectomy [14, 19]. In this study, we did not design to collect most of these measurements. In our opinion, the addition of these measurements could have significantly weakened other data quality since the patient becomes already too stressed prior to surgery. In the study protocol, we considered how the patient rated their trust in their surgeon and their surgeon’s BCS recommendation as two key actionable factors. We believed both measurements were the most representative of the interaction between the patient and their surgeon on the BCS recommendation and choice.

There are a few surgeon-related factors identified influencing the BCS choice [2, 8, 20]. A large review on the Surveillance, Epidemiology, and End Results (SEER) database (*N* =56,768) concluded that female surgeons had a higher rate of BCS (OR =1.40, *p*<0.05) [20]. Surgeons who graduated in “*more recent years*,” performed “*more BCS procedures*,” and had “*academic affiliation*” were more likely to recommend and carry out BCS [2, 8]. To control for gender (we only had one female surgeon among 11 participating surgeons in total) and practice years, a BCS-CI score scale was created to adjust for their effects in this study.

Both consistent and inconsistent findings with other studies are identified in this study. Residence status and age (25–75 years) were found to be insignificant influential factors of BCS. However, such findings were inconsistent with some published studies [7, 10]. We considered that changes in Chinese patients’ attitudes to and acceptance of BCS over the last two decades in mainland China might have contributed to this observation. Compared to tumor sizes ≥ 3 cm, sizes <2 and 2–2.9 cm were significantly associated with the BCS procedure with ORs of 3.792 (*p*<0.001) and 2.260 (*p*=0.001), respectively. Other studies had similar findings [8, 10, 19]. In this study, the average tumor size of Chinese BCS candidates (*n*=1050) and procedure patients (*n* = 202) were 2.4 ± 0.9 cm and 2.0 ± 0.8 cm, respectively. We noticed that both statistics were remarkably higher than the mean tumor size (1.5 cm) of BCS procedure patients in the US [20]. Compared to patients with a “*middle and under*” education, patients with a “*high school*” and “*college and above*” education in this study were found more likely to have BCS with ORs of 1.775–1.875 (both *p*<0.030). Indeed, high socioeconomic status and its indicators like education or income or urban living were often reported to be associated with high rates of BCS choice [5, 21]. While the exact mechanisms behind these associations above are considered to be complex and require further investigation, we believe that considering these specific demographics would help target candidates for BCS among Chinese women.

It is hard to fully explain why Chinese women with EBC in mainland China have a low BCS rate. Compared to other races, Chinese and Asian/Pacific Islander women were reported to be more likely to undergo mastectomies [21–23]. We considered the possible reasons as the following: Chinese women with EBC (1) would have less cosmetic gain from the BCS due to their relatively smaller breast sizes, (2) are more concerned about hair, weight, and facial appearances, which are often regarded to be the main determinants of social identity, and (3) are less worried about the detrimental effect on sexuality and marriage from the breast loss [23–25]. Except for the tumor size, these factors were not designed to be investigated in the study.

This study was designed to quantitatively characterize the pre-operative surgeon-patient dialog and profile them with the BCS choice. We considered the dialog quality as the result of actionable elements. Although the “*trust on surgeon*” and “*BCS recommendation*” strengths were found to be related to each other (chi-squared =54.6, *p*<0.001), they were independently associated with the BCS procedure at a significant level (*p*<0.050) regardless of age, residence, and other factors. Supported by the medical decision-making theories, we believe that cultivating the patient’s trust would be the start of an effective BCS recommendation from a surgeon. In fact, some published studies supported this review and have found that stronger trust and firmer recommendation were significantly affecting a patient’s choice of BCS instead of a mastectomy [26–28]. The race-sensitive medical communication between a patient and her surgeon should be more flexible and oriented towards evidence-based race-related sociocultural diversity [29]. How to effectively improve the pre-operative surgeon-patient dialog should be studied further.

### Study strengths and limitations

This study had many strengths. First, it was a large prospective study with many factors analyzed. Only less than 4% of enrolled patients were excluded due to an interview absence or/and an involuntary BCS-to-mastectomy switch. Second, a stepwise model building strategy from many variables related to tumor, patient, and surgeon was used. Third, the stratified analyses by age or residence enhanced the conclusions. Fourth, the “*trust on surgeon*” and “*BCS recommendation*” strengths in Likert scales were analyzed as two independent composite variables in measuring the surgeon-patient dialog. Lastly, the questionnaires used in this study have demonstrated excellent internal consistencies for the “*trust on surgeon*” (Cronbach α = 0.84) and “*BCS recommendation*” (Cronbach α = 0.81) strength subscales.

This study has some limitations. The confidential data of marital status, family/individual income, and relationship with husband/caregiver were not collected. The effect of the additional RT cost and breast reconstruction option on the BCS procedure was not evaluated. The surgeons were not randomly assigned to the enrolled patients. The inconsistency of BCS recommendation strength assessed by the patient and her surgeon was possible, and could not be reliably investigated given the lack of quality chart note data. Additionally, the specific measurements of physical appearance, sexuality, attraction, feminist, and psychological aspects were not collected. Lastly, the BCS rates in this study should not be simply compared with those in Europe or USA given the different criteria for BCS and calendar years. Nonetheless, these limitations should not have significantly affected the validity of our study conclusions.

### Clinical implications

The strong positive relationships of the “*trust on surgeon*” and “*BCS recommendation*” with the BCS in Chinese patients with EBC are well-established in this large prospective cohort study. In order to promote BCS among Chinese women with EBC, stronger trust and recommendation of BCS should be encouraged between a surgeon and their patient.

## Conclusions

Independently from tumor size and others, the stronger trust on the surgeon and BCS recommendation by the surgeon are associated with a higher rate of BCS in Chinese women with EBC. Interventional trials are needed to confirm this finding.

## Data Availability

The data that support the findings of this study are available from the corresponding authors upon reasonable request and with the permission of the Fourth Hospital of Hebei Medical University.

## Abbreviations

ALND: Axillary lymph node dissection
BCS: Breast-conserving surgery
BCS-CI: BCS Composite Index
CI: Confidence interval
DCIS: Ductal carcinoma in situ
EBC: Early breast cancer
LN: Lymph node
MRI: Magnetic resonance imaging
NAC: Neoadjuvant chemotherapy
OR: Odd ratio
QoL: Quality of life
RT: Radiotherapy
SD: Standard deviation
SLNB: Sentinel lymph node biopsy
